# The physiological effect of early pregnancy on a woman’s response to a submaximal cardiopulmonary exercise test

**DOI:** 10.14814/phy2.14624

**Published:** 2020-11-15

**Authors:** Rianne C. Bijl, Jérôme M. J. Cornette, Kim van der Ham, Merle L. de Zwart, Dinis Dos Reis Miranda, Régine P. M. Steegers‐Theunissen, Arie Franx, Jeroen Molinger, M. P. H. (Wendy) Koster

**Affiliations:** ^1^ Department of Obstetrics and Gynecology Erasmus MC University Medical Centre Rotterdam The Netherlands; ^2^ Department of Intensive Care Adults Erasmus MC University Medical Centre Rotterdam The Netherlands; ^3^ Department of Anesthesiology & Intensive Care Medicine Human Physiology and Pharmacology Lab (HPPL) Duke University Medical Centre Durham NC USA

**Keywords:** breath‐by‐breath analysis, cardiopulmonary exercise test, impedance cardiography, pregnancy, ventilatory threshold

## Abstract

Given all its systemic adaptive requirements, pregnancy shares several features with physical exercise. In this pilot study, we aimed to assess the physiological response to submaximal cardiopulmonary exercise testing (CPET) in early pregnancy. In 20 healthy, pregnant women (<13 weeks gestation) and 20 healthy, non‐pregnant women, we performed a CPET with stationary cycling during a RAMP protocol until 70% of the estimated maximum heart rate (HR) of each participant. Hemodynamic and respiratory parameters were non‐invasively monitored by impedance cardiography (PhysioFlow^®^) and a breath‐by‐breath analyzer (Oxycon^TM^). To compare both groups, we used linear regression analysis, adjusted for age. We observed a similar response of stroke volume, cardiac output (CO) and HR to stationary cycling in pregnant and non‐pregnant women, but a slightly lower 1‐min recovery rate of CO (−3.9 [−5.5;‐2.3] vs. −6.6 [−8.2;‐5.1] L min^−1^ min^−1^; *p* = .058) and HR (−38 [−47; −28] vs. −53 [−62; −44] bpm/min; *p* = .065) in pregnant women. We also observed a larger increase in ventilation before the ventilatory threshold (+6.2 [5.4; 7.0] vs. +3.2 [2.4; 3.9] L min^−1^ min^−1^; *p* < .001), lower P_ET_CO_2_ values at the ventilatory threshold (33 [31; 34] vs. 36 [34; 38] mmHg; *p* = .042) and a larger increase of breathing frequency after the ventilatory threshold (+4.6 [2.8; 6.4] vs. +0.6 [−1.1; 2.3] breaths min^−1^ min^−1^; *p* = .015) in pregnant women. In conclusion, we observed a slower hemodynamic recovery and an increased ventilatory response to exercise in early pregnancy.

## Graphical abstract

The response to a cardiopulmonary exercise test is already altered in the first trimester of pregnancy. Pregnant women show a slower hemodynamic recovery and an excessive ventilatory response to submaximal exercise.

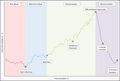

## 1 INTRODUCTION

Human pregnancy initiates essential physiological changes in the mother in order to meet the increased demands of the growing placenta and fetus. Almost all maternal organ systems require such changes, including the cardiovascular, gastrointestinal, renal, and respiratory systems. These changes start very early in pregnancy. Cardiovascular adaptation in the first trimester is reflected by a decrease of 35%–40% in systemic vascular resistance and a subsequent increase of 8% in the left ventricular stroke volume (Cornette et al., 2014). Pulmonary adaptation to pregnancy also starts in the first weeks of pregnancy, with a rise in resting minute ventilation (V_E_) up to 20%–50% at term compared to the non‐pregnant state (Clapp et al., 1988; Hegewald & Crapo, 2011).

Hence, pregnancy can be viewed as a stress test for the mother, which most mothers pass without clinical problems. However, it is thought that, in women who develop placenta‐related pregnancy complications, such as preeclampsia and fetal growth restriction, an increased risk for cardiovascular disease is unmasked by the stressed state of pregnancy (Bamfo et al., 2008; Barker & Thornburg, 2013; Sattar & Greer, 2002).

A cardiopulmonary exercise test (CPET) is used to functionally assess the integrative exercise response of the cardiovascular, respiratory, and peripheral muscular systems. Since exercise mimics a state of systemic metabolic stress, abnormal functions can be revealed that otherwise would have remained undetected during measurements at rest. In a recently published meta‐analysis, describing the effect of exercise interventions during pregnancy, the authors state there is a lack of information on exercise and the measures of cardiorespiratory fitness during pregnancy, since historically multiple different exercise protocols with different outcome measures have been used (Cai et al., 2020). Moreover, previous studies showed differences in the response to exercise between pregnant women and non‐pregnant women, but were predominantly executed in the second and third trimester of pregnancy under varying exercise protocols (Aardenburg et al., 2006; Jaque‐Fortunato et al., 1996; Jensen et al., 2010; McAuley et al., 2005; Pivarnik et al., 1991; Weissgerber et al., 2006). Knowledge of the respiratory response to exercise in early pregnancy is still limited and the hemodynamic response to CPET has rarely been described during the first trimester (Spatling et al., 1992). Since placenta‐related pregnancy complications originate in early pregnancy, this period is particularly important for early identification of such complications and therefore, possibilities to prevent these. However, before we can evaluate the predictive value of cardiorespiratory parameters during CPET in early pregnancy, we need to determine the normal response in healthy, pregnant women (Meah et al., 2018).

Given the known adaptive requirements of a woman's respiratory, cardiovascular, and metabolic system during pregnancy and—based on previous research—the response to exercise during pregnancy, we hypothesize that the response to CPET is altered during (early) pregnancy. The aim of this pilot study was to assess differences in response to hemodynamic and respiratory parameters between healthy, pregnant women in their first trimester of pregnancy and healthy, non‐pregnant women during CPET.

## 2 MATERIALS AND METHODS

### 2.1 Ethical approval

The study protocol was approved by the Medical Ethics Committee of the Erasmus MC, Rotterdam, the Netherlands (MEC‐2018‐080). All participants provided written, informed consent, and the study protocol conformed to the standards set by the Declaration of Helsinki.

### 2.2 Study design and population

Between May 2018 and August 2018, we performed a cross‐sectional assessment of 20 pregnant women (singleton pregnancies, gestational age < 13 weeks) who were recruited from the outpatient clinic of the Department of Obstetrics and Gynecology of the Erasmus MC, University Medical Center Rotterdam, the Netherlands and 20 non‐pregnant women from personal networks. Exclusion criteria at the time of recruitment for both groups included: any known pre‐existing cardiovascular, respiratory, hypertensive or systemic disorder, a history of adverse pregnancy outcomes (i.e., preeclampsia, pregnancy‐induced hypertension, and intra‐uterine fetal growth restriction), multiple pregnancies, women who smoke or quit smoking less than 3 months ago.

### 2.3 Study procedures

#### 2.3.1 Baseline characteristics

Prior to CPET, all women were asked to complete a short questionnaire regarding their age, parity, and level of physical activity (exercising or sedentary). Pregnant women were also asked to provide information about the mode of conception and duration of the pregnancy (i.e., gestational age). Mode of conception was categorized as pregnancies conceived by hormonally assisted reproductive techniques (in vitro fertilization with or without intra‐cytoplasmic sperm injection or intra‐uterine insemination with ovulation induction) or pregnancies conceived within a natural cycle (spontaneous conception or cryopreserved embryo transfer). For all women, weight and height were measured at baseline assessment and used to calculate the current body mass index (BMI). Additionally, an automated blood pressure measurement was performed on the left arm.

##### CPET

**FIGURE 1:**
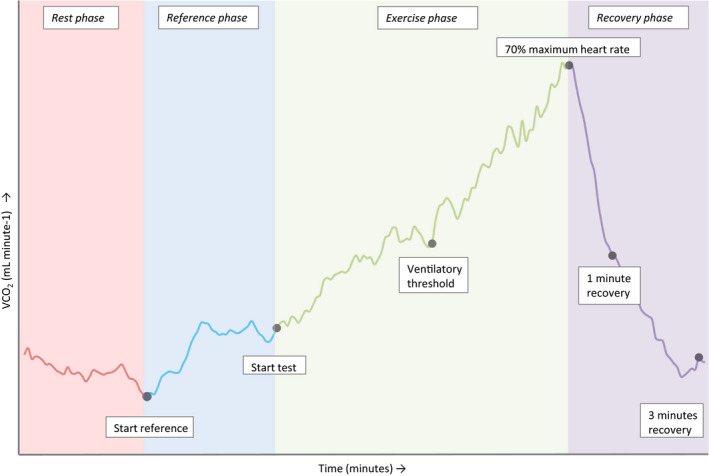
The output during CPET for one respiratory parameter (VCO_2_) as an example to illustrate the four test phases and the six different time points

All participants performed one submaximal CPET until 70% of their estimated maximal heart rate (HR), which is considered safe for both mother and fetus during pregnancy (Larsson & Lindqvist, 2005; Meah et al., 2018). The estimated maximal HR was calculated using the Tanaka formula: 208 – (0.7 × age; Tanaka et al., 2001). The CPET was performed on an upright cycle ergometer (Ergometer ergometrics 800S, ergoline GmbH) in which the load could increase stepwise and manually. All measurements were performed according to standardized protocols with the participant in sitting position on the cycle ergometer, during four different test phases (Figure 1):

1. Rest phase: retrieval of baseline measurements for three minutes.
2. Reference phase: cycling on the unloaded cycle ergometer for three minutes at a speed of 40 revolutions per minute (rpm).
3. Exercise phase: cycling at a speed of 60–70 rpm during a RAMP protocol (start load 25 Watt followed by a rise of 5 Watt in resistance every 12 s automatically) until 70% of the estimated maximum HR was reached.
4. Recovery phase: 3 min of rest.

If the participant experienced any discomfort, like dizziness or pain, or if the oxygen saturation was below 94%, the test was stopped. In pregnant participants, a transabdominal ultrasound scan was performed both before and after the CPET protocol to confirm pregnancy viability.

#### 2.3.2 Hemodynamic monitoring

For monitoring of the hemodynamic parameters, we used signal morphology impedance cardiography (SM‐ICG) by PhysioFlow^®^ (Manatec Biomedical; Dupuis et al., 2018). ICG uses impedance variations, induced by cardiac flow, in a low‐magnitude, high‐frequency, alternating current, which is transmitted through the chest to calculate SV. SM‐ICG additionally filters all noise artifacts (e.g., by moving or breathing) in the impedance signal that cannot be related to the cardiac cycle or cardiac blood flow. This provides a stable signal and therefore a non‐invasive continuous monitoring method. SM‐ICG is suited to monitor trends over time and therefore is one of the very few methods technically permitting continuous and nearly instantaneous, operator‐independent, non‐invasive monitoring of changes in SV and cardiac output (CO) during exercise.

PhysioFlow^®^ was connected with two disposable electrodes on the neck, two on the back and two on the chest, which detect and transmit electrical and impedance changes in the thorax. PhysioFlow^®^ was used according to the manufacturer's guide for exercise testing, with a semi‐continuous output every 5 s providing information on the participants’ CO, HR, and SV.

#### 2.3.3 Respiratory monitoring

To determine the breath‐by‐breath metabolic response to exercise, we used the Oxycon^TM^ Mobile device (VIASYS Healthcare GmbH). This is a portable system with a facemask that collects breath‐by‐breath data which is wirelessly transferred to a host computer system. Calibration of the breath‐by‐breath analyzer was performed prior to each exercise test, according to the manufacturer's manual. Next, spirometry was performed during which the participants were asked to inhale to their maximal volume, followed by a powerful and complete exhale with the breath‐by‐breath mask over their nose and mouth to determine the maximal voluntary ventilation.

During each expiration, the tidal volume (Vt), volume of oxygen (O_2_), volume of carbon dioxide (CO_2_), and breathing frequency (BF) were registered. Based on these measurements and the volume of O_2_ in ambient air, the following parameters were derived: minute ventilation (V_E_), O_2_ uptake (VO_2_), CO_2_ elimination (VCO_2_), end‐tidal partial pressure of O_2_ (P_ET_O_2_), end‐tidal partial pressure of CO_2_ (P_ET_CO_2_) and the ventilatory equivalents for VO_2_ and VCO_2_ (EqO_2_; EqCO_2_).

### 2.4 Outcome parameters

The primary endpoint was the difference in response of CO during CPET, measured with SM‐ICG, between pregnant and non‐pregnant women. This endpoint was chosen for CO rises during exercise due to the increased metabolic demands of the peripheral muscular system. As mentioned before, pregnancy is associated with the elevation of the systemic demands in rest and therefore, we hypothesize that pregnant women will show a different CO adaptation pattern to exercise (Butte & King, 2005). SM‐ICG is used to continuously measure CO in an operator‐independent way, with high repeatability making it suitable for monitoring during a state of exercise (Bijl, Valensise, et al., 2019; Staelens et al., 2016).

To evaluate and compare the response of CO to exercise, we report on its relative (%) changes during the reference phase, exercise phase, 1 min of recovery, and 3 min of recovery (Figure 1), relative to resting values. To adjust for the duration of each test phase, the slopes of CO response during the exercise phase and during the 1 min of recovery were calculated. Hereby, the slope represents the rate of change per minute. By reporting on relative changes and slopes of CO derived by SM‐ICG, the effect of potential over‐ or underestimation of absolute measured values is eliminated.

Secondary outcome parameters were defined as the difference in response of HR and SV, assessed similarly as CO, and differences in the respiratory response to exercise. The respiratory response to exercise is reported on by comparing absolute values of V_E_, BF, V_t_, VO_2_, VCO_2_, P_ET_O_2_, P_ET_CO_2_, EqO_2_, and EqCO_2_ at six different time points (Figure 1): “start reference,” “start test,” “ventilatory threshold,” “70% maximum heart rate,” “1 min recovery,” and “3 min recovery.” Also, slopes of the respiratory parameters during the exercise phase before the ventilatory threshold, the exercise phase after the ventilatory threshold, and during the 1 min of recovery were calculated. In classical CPET assessment, the oxygen pulse slope, derived from breath‐by‐breath analysis, is accepted as a reflection of SV. Therefore, oxygen pulse slopes will be reported for comparison with SV and CO slopes derived from PhysioFlow.

The ventilatory threshold was defined as the point during exercise where aerobic energy production is supplemented with anaerobic mechanisms. The ventilatory threshold is considered a reliable submaximal parameter and is strongly correlated with outcomes during a CPET until exhaustion, such as maximal VO_2_ (Kunutsor et al., 2017). This time point was independently determined per participant by two researchers (RCB and MLdZ) using the so‐called equivalents method. With this method, EqO_2_ and EqCO_2_ are plotted against VO_2_. The point where EqO_2_ increases, yet before EqCO_2_ starts to increase, corresponds with the ventilatory threshold (Levett et al., 2018).

In addition, we assessed the Oxygen Uptake Efficiency Slope (OUES) by calculating the slope between VO2 and the logarithmically transformed VE and the VE/VCO2 slope by dividing VE by VCO2; both from the start of the exercise phase to the ventilatory threshold. These slopes are indicators of respiratory efficiency during submaximal exercise testing (Hollenberg & Tager, 2000; Sun et al., 2002).

### 2.5 Data analysis

Individual PhysioFlow^®^ and Oxycon^TM^ Mobile datasets were processed in Microsoft Excel and parameters were averaged over 20 s surrounding each of the six specific time points (i.e., 10 s before and 10 s after the time point). In the Oxycon^TM^ Mobile datasets, a 5‐s moving average was used for each parameter to reduce the breath‐by‐breath variability (Levett et al., 2018; Robergs et al., 2010).

Statistical analyses were performed using SPSS package 24 (IBM SPSS Statistics). Data were visualized in Q‐Q plots in order to evaluate distributions, which were all normal. Participant characteristics were expressed as means ± standard deviation (*SD*) or numbers (percentage) and compared between pregnant and non‐pregnant women using linear regression analysis for continuous variables and Chi‐square tests for categorical variables.

Means of relative changes and slopes of the hemodynamic parameters and absolute values and slopes of the respiratory parameters were compared between pregnant and non‐pregnant women, using generalized linear regression models. Pearson's correlation coefficients were used to identify potential confounders in our dataset; only age significantly affected the relationship and therefore, all analyses were adjusted for age. BMI was not identified as a potential confounder. However, we also report VO_2_ in mL/kg/min, hereby providing a crude weight correction. Throughout the manuscript, only age‐adjusted, estimated marginal means with accompanying 95% confidence intervals (CI) are reported. Results for all comparisons were considered statistically significant if *p*‐values were <.05.

## 3 RESULTS

### 3.1 Study participants

A total of 20 healthy, pregnant women in their first trimester of pregnancy were included and 20 healthy, non‐pregnant women participated in the control group. The baseline characteristics of the participants are summarized in Table 1. The pregnant women were significantly older (33.7 ± 4.3 vs. 25.3 ± 1.9 years; *p*=<.001), had a higher BMI (25.9 ± 5.4 vs. 22.3 ± 2.5 kg/m^2^; *p* = .030), and were less often physically active (35% vs. 70%, *p* = .069) compared to the non‐pregnant women. Of the pregnant women, 14 women (70%) were nulliparous, while in the non‐pregnant group 19 women (95%) were nulliparous (*p* = .037). Of the 20 pregnancies studied, 13 (65%) were conceived after hormonally assisted reproductive techniques. The overall mean gestational age was 11 weeks and 1 day ± 1 week and 2 days.

**TABLE 1:** Data are presented as means ± *SD* and numbers %

	Pregnant	Non‐pregnant	*p*‐value
	*n = 20*	*n = 20*	
Age (years)	33.7 ± 4.3	25.3 ± 1.9	<.001
BMI (kg/m^2^)	25.9 ± 5.4	22.3 ± 2.5	.03
Physical activity level			
Exercising	7 (35%)	14 (70%)	.069
Sedentary	12 (60%)	6 (30%)	
Unknown	1 (5%)	0 (0%)	
Parity			
Nulliparous	14 (70%)	19 (95%)	.037
Multiparous	6 (30%)	1 (5%)	
Conception mode			
Hormonally assisted	13 (65%)	—	NA
Natural cycle	7 (35%)	—	
Gestational age (weeks^+days^)	11 ^+ 1^ ± 1 ^+2^	—	NA
Heart rate in rest (bpm)	87 ± 9.2	82 ± 8.5	.113
Systolic blood pressure (mmHg)	109 ± 10.1	111 ± 11.4	.718
Diastolic blood pressure (mmHg)	66 ± 7.7	67 ± 7.9	.968

Abbreviations: BMI, body‐mass index; bpm, beats per minute; kg, kilogram; m^2^, cubic meter; mmHg, millimeters of mercury; NA, not applicable.

At baseline, there were no significant differences in HR (87 ± 9.2 bpm vs. 82 ± 8.5 bpm, *p* = .113) and blood pressure (systolic 109 ± 10.1 mmHg vs. 111 ± 11.4 mmHg, *p* = .718; diastolic 66 ± 7.7 mmHg vs. 67 ± 7.9 mmHg, *p* = .968) between pregnant and non‐pregnant women.

### 3.2 Hemodynamic parameters

#### 3.2.1 Reference phase

While cycling on the unloaded ergometer, no significant differences in relative changes of CO and SV were observed between pregnant women and non‐pregnant women. Only a small decrease of 3% in HR was observed in the pregnant women, against an increase of 6% in HR in the non‐pregnant women (*p* = .035).

#### 3.2.2 Exercise phase

**FIGURE 2:**
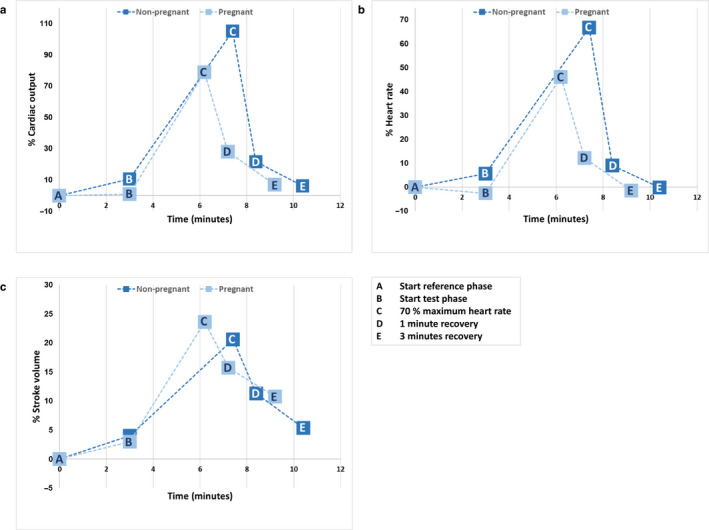
Response of hemodynamic parameters cardiac output, heart rate, and stroke volume depicted as age‐adjusted means of relative (%) changes in pregnant and non‐pregnant women during cardiopulmonary exercise testing

The time to reach 70% maximum heart rate was shorter in pregnant women compared to non‐pregnant women, although this was not statistically significant after adjustment for age (3.2 ± 1.0 vs. 4.4 ± 1.4 min, *p* = .150; Figure 2).

In the pregnant women, a trend toward less increase of CO was observed compared to the non‐pregnant women (+79% [95% CI 60; 98] vs. +105% [95% CI 86; 124, *p* = .135; Table 2a; Figure 2). This was related to a smaller percentage increase in HR (+46% [95% CI 36; 56] vs. +67% [95% CI 58; 76]; *p* = .019) with a similar percentage increase in SV (+24% [95% CI 13; 34] vs. +21% [95% CI 10; 31]; *p* = .719) in the pregnant women compared to the non‐pregnant women.

**TABLE 2a:** Data are presented as estimated marginal means (*µ*) with a 95% confidence interval (CI) after age‐adjustment

	Reference phase					Exercise phase					Recovery phase (1 min)					Recovery phase (3 min)				
	P		NP		*p*‐value	P		NP		*p*‐value	P		NP		*p*‐value	P		NP		*p*‐value
	*µ*	95% CI	*µ*	95% CI		*µ*	95% CI	*µ*	95% CI		*µ*	95% CI	*µ*	95% CI		*µ*	95% CI	*µ*	95% CI	
% Cardiac output	+1	−9; 10	+11	1; 20	.244	+79	60; 98	+105	86; 124	.135	+28	13; 43	+22	7; 36	.609	+7	−3; 17	+6	−4; 16	.959
% Heart rate	**−3**	**−7; 1**	**+6**	**1; 10**	**.035**	**+46**	**36; 56**	**+67**	**58; 76**	**.019**	+12	4; 21	+9	1; 17	.667	−1	−7; 4	−1	−6; 5	.906
% Stroke volume	+3	−4; 10	+4	−3; 11	.835	+24	13; 34	+21	10; 31	.719	+16	6; 26	+11	2; 21	.614	+11	3; 19	+5	−2; 13	.466

Abbreviations: P, pregnant; NP, non‐pregnant.

Bold indicates statistical significant values.

The slopes of CO, HR, and SV during the exercise phase were similar for the pregnant and non‐pregnant women (Table 2b). CO increased with 1.4 L min^−1^ min^−1^ in the pregnant women, compared to 1.7 L min^−1^ min^−1^ in the non‐pregnant women (*β* −0.276, *p* = .343). HR increased with 13 bpm/min in both groups (*β* 0.003, *p* = .993) and SV with 3.4 ml/min in the pregnant women versus 4.4 ml/min in the non‐pregnant women (*β* −0.181, *p* = .537). In the pregnant women, the oxygen pulse slope was on average 1.25 ± 0.39 ml beat^−1^ min^−1^ compared to 1.16 ± 0.33 ml beat^−1^ min^−1^ in the non‐pregnant women (*p* = .449).

**TABLE 2b:** Data are presented as estimated marginal means (*µ*) with a 95% confidence interval and regression coefficients (*β*) after adjustment for age

	Exercise phase						Recovery phase (1 min)					
	P		NP		*β*	*p*‐value	P		NP		*β*	*p*‐value
	*µ*	95% CI	*µ*	95% CI			*µ*	95% CI	*µ*	95% CI		
CO (L min^−1^ min^−1^)	1.4	1.0; 1.7	1.7	1.3; 2.0	−0.276	.343	−3.9	−5.5; −2.3	−6.6	−8.2; −5.1	0.530	.058
HR (bpm/min)	13	10; 15	13	10; 15	0.003	.993	−38	−47; −28	−53	−62; −44	0.537	.065
SV (ml/min)	3.4	1.5; 5.2	4.4	2.6; 6.3	−0.181	.537	−6.2	−14.0; 1.6	−10.6	−18.1; −3.1	0.181	.540

Abbreviations: bpm, beats per minute; CO, cardiac output; HR, heart rate; L, liter; min, minute; mL, millilitre; NP, non‐pregnant; P, pregnant; SV, stroke volume.

#### 3.2.3 Recovery phase

One minute after the exercise phase ended, the hemodynamic parameters of neither one of the groups had returned to their baseline values (Table 2a; Figure 2). There were no differences in relative decrease during the recovery phase between the pregnant and non‐pregnant women. However, during one minute of recovery, pregnant women showed a less steep CO recovery slope (−3.9 vs. −6.6 L min^−1^ min^−1^; *β* 0.530, *p* = .058), a less steep HR recovery slope (−37.6 vs. −52.9 bpm/min; *β* 0.537, *p* = .065) and a similar SV recovery slope (−6.2 vs. −10.6 ml/min; *β* 0.181, *p* = .540) compared to the non‐pregnant women (Table 2b).

After three minutes of rest, CO had almost returned to resting values (7% vs. 6%; *p* = .959) in both the pregnant and non‐pregnant women. SV in the pregnant women was still 11% above their resting value versus 5% in the non‐pregnant women (*p* = .466; Table 2a, Figure 2).

### 3.3 Respiratory parameters

#### 3.3.1 Reference phase

**FIGURE 3:**
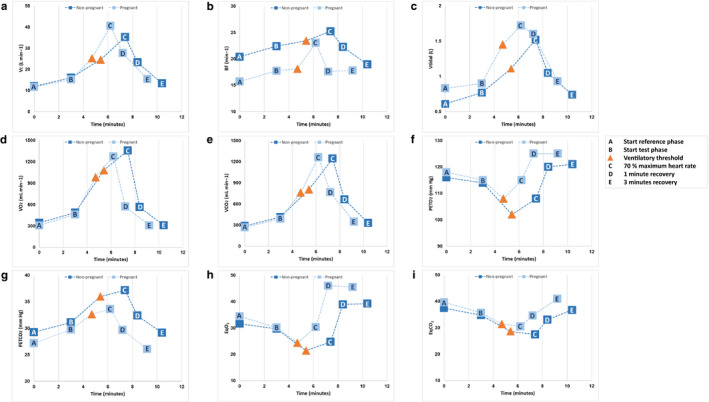
Response of respiratory parameters depicted as age‐adjusted means of pregnant and non‐pregnant women during cardiopulmonary exercise testing. (a) V_E_ = minute ventilation in liter min^−1^; (b) BF = breathing frequency per minute; (c) V_tidal_ = tidal volume in litres; (d) VO_2_ = oxygen consumption in milliliter per minute; (e) VCO_2_ = carbon dioxide production in milliliter per minute; (f) P_ET_O_2_ = end‐tidal pressure of oxygen in millimeters of mercury; (g) P_ET_CO_2_ = end‐tidal pressure of carbon dioxide in millimeters of mercury; (h) EqO_2_ = equivalent of oxygen; (i) EqCO_2_ = equivalent of carbon dioxide

At the start of the reference phase, there were no differences in V_E_, VCO_2_, P_ET_O_2_, P_ET_CO_2_, EqO_2_ or EqCO_2_ between groups after adjusting for age (Table 3a; Figure 3). However, the pregnant women had a lower BF (16 [95% CI 13; 18] vs. 20 [95% CI 18; 23] breaths min^−1^; *p* = .028) and a higher V_t_ (0.8 [95% CI 0.7; 1.0] vs. 0.6 [95% CI 0.5; 0.7] liter; *p* = .047) compared to the non‐pregnant women. Also, VO_2_ in the pregnant women was lower compared to the non‐pregnant women (4.1 [95% CI 3.4; 4.9] vs. 5.6 [95% CI 4.9; 6.3] ml kg^−1^ min^−1^; *p* = .027) when expressed in mL/kg/min, but not when expressed in ml/min.

**TABLE 3a:** Data are presented as estimated marginal means (*µ*) with a 95% confidence interval after adjustment for age

	Start reference					Start test					Ventilatory threshold					70% maximum heart rate					1 min recovery					3 min recovery				
	P		NP		*p*‐value	P		NP		*p*‐value	P		NP		*p*‐value	P		NP		*p*‐value	P		NP		*p*‐value	P		NP		*p*‐value
	*µ*	95% CI	*µ*	95% CI		*µ*	95% CI	*µ*	95% CI		*µ*	95% CI	*µ*	95% CI		*µ*	95% CI	*µ*	95% CI		*µ*	95% CI	*µ*	95% CI		*µ*	95% CI	*µ*	95% CI	
V_E_ (L/min)	12	10; 13	12	11; 14	.829	15	13; 17	16	14; 18	.618	25	22; 28	25	22; 28	.815	41	34; 47	35	29; 41	.339	28	24; 32	23	20; 27	.220	15	13; 18	13	11; 16	.380
BF (breaths/min)	**16**	**13; 18**	**20**	**18; 23**	**.028**	**18**	**16; 20**	**22**	**20; 24**	**.013**	**18**	**16; 21**	**23**	**21; 26**	**.025**	23	20; 26	25	23; 28	.361	**18**	**15; 20**	**22**	**20; 24**	**.017**	18	16; 20	19	17; 21	.573
V_t_ (L)	**0.8**	**0.7; 1.0**	**0.6**	**0.5; 0.7**	**.047**	0.9	0.8; 1.0	0.8	0.6; 0.9	.263	1.5	1.2; 1.7	1.1	0.9; 1.3	.087	1.7	1.5; 2.0	1.5	1.3; 1.7	.221	**1.6**	**1.4; 1.8**	**1.0**	**0.8; 1.2**	**.001**	0.9	0.8; 1.1	0.7	0.6; 0.9	.121
VO_2_ (ml/min)	307	263; 352	344	301; 387	.358	461	406; 516	488	435; 540	.589	983	873; 1,094	1,082	976; 1,189	.326	1,276	1,117; 1,435	1,358	1,204; 1511	.579	573	509; 637	566	509; 623	.887	308	257; 359	309	262; 356	.983
VO_2_ (ml kg^−1^ min^−1^)	**4.1**	**3.4; 4.9**	**5.6**	**4.9; 6.3**	**.027**	**6.2**	**5.6; 6.9**	**8.0**	**7.3; 8.6**	**.006**	**13.4**	**11.6; 15.1**	**17.6**	**15.9; 19.2**	**.010**	**17**	**15; 20**	**22**	**20; 25**	**.027**	7.8	6.9; 8.7	9.2	8.4; 10	.072	4.3	3.4; 5.3	5	4.1; 5.8	.445
VCO_2_ (ml min^−1^ min^−1^)	268	231; 304	287	252; 323	.551	391	342; 440	414	367; 462	.604	762	658; 866	805	705; 905	.653	1,264	1,073; 1,454	1,245	1,061; 1,429	.902	766	657; 874	665	569; 762	.273	348	283; 413	329	269; 389	.718
P_ET_O_2_ (mmHg)	118	116; 120	116	114; 118	.143	115	113; 117	114	112; 115	.473	**108**	**106; 111**	**102**	**100; 105**	**.008**	**115**	**112; 118**	**108**	**105; 111**	**.018**	**125**	**122; 127**	**120**	**117; 122**	**.022**	125	122; 128	121	118; 123	.070
P_ET_CO_2_ (mmHg)	27	27; 29	29	28; 31	.153	30	28; 31	31	30; 33	.306	**33**	**31; 34**	**36**	**34; 38**	**.042**	34	32; 36	37	35; 39	.051	30	28; 32	32	31; 34	.140	**26**	**24; 28**	**29**	**28; 31**	**.047**
EqO_2_	34	32; 37	32	29; 34	.244	30	28; 32	30	28; 32	.793	**24**	**23; 26**	**21**	**20; 23**	**.004**	**30**	**28; 32**	**25**	**23; 27**	**.003**	46	42; 51	39	35; 43	.072	46	41; 50	39	35; 43	.101
EqCO_2_	40	37; 42	37	35; 40	.366	36	33; 38	35	33; 37	.661	31	30; 33	29	27; 30	.065	**31**	**29; 32**	**28**	**26; 29**	**.032**	35	32; 37	33	31; 35	.415	41	38; 44	37	34; 39	.077

Abbreviations: BF, breathing frequency; EqCO_2_, equivalent of carbon dioxide; EqO_2_, equivalent of oxygen; L, liter; min, minute; ml, milliliter; mmHg, millimetres of mercury; NP, non‐pregnant; P, pregnant; P_ET_CO_2_, end‐tidal pressure of carbon dioxide; P_ET_O_2_, end‐tidal pressure of oxygen; VCO_2_, carbon dioxide production; V_E_, minute ventilation; VO_2_, oxygen consumption; Vt, tidal volume.

Bold indicates statistical significant values.

#### 3.3.2 Exercise phase

Although not statistically significant after adjustment for age, the time to reach the ventilatory threshold (1.65 ± 0.7 vs. 2.36 ± 0.7 min, *p* = .094) was also shorter in pregnant women compared to non‐pregnant women (Figure 3).

At the ventilatory threshold, VO_2_ was 13.4 [95% CI 11.6;15.1] ml kg^−1^ min^−1^ in the pregnant women versus 17.6 [95% CI 15.9;19.2] ml kg^−1^ min^−1^ in the non‐pregnant women; *p* = .010), there were no differences in means of V_E_, VO_2_ (ml/min) and VCO_2_ between groups (Table 3a; Figure 3). In pregnant women, the lower BF values remained with slightly higher V_t_ values (1.5 [95% CI 1.2; 1.7] vs. 1.1 [95% CI 0.9; 1.3] liter; *p* = .087) compared to the non‐pregnant women. Moreover, P_ET_CO_2_ was lower and P_ET_O_2_, EqO_2_, and EqCO_2_ were higher in the pregnant women compared to the non‐pregnant women.

At the end of the exercise phase at 70% maximum HR, peak VO_2_ was 17.1 [95% CI 14.6;19.6] ml kg^−1^ min^−1^ in the pregnant women versus 22.3 [95% CI 19.8; 24.7] ml kg^−1^ min^−1^ in the non‐pregnant women (*p* = .027). There were no differences in means of V_E_, VO_2_ (ml/min) and VCO_2_, BF, and V_t_ between groups (Table 3a; Figure 3). In pregnant women, P_ET_CO_2_ remained lower and P_ET_O_2_, EqO_2_, and EqCO_2_ remained higher compared to non‐pregnant women.

During the exercise phase before the ventilatory threshold, both the increase in V_E_ (6.2 vs. 3.2 L min^−1^ min^−1^; *β* 0.861, *p* < .001) and the increase in V_t_ (0.4 vs. 0.1 L/min; *β* 0.893, *p* < .001) were higher in pregnant women compared to those in non‐pregnant women (Table 3b). The same was true for the increase in VO_2_ (332 vs. 244 ml min^−1^ min^−1^; *β* 0.581, *p* = .028) and the decrease in VCO_2_ (227 vs. 147 ml min^−1^ min^−1^; *β* 0.794, *p* = .001). After the ventilatory threshold, the only difference regarding slopes was observed for BF; its increase was higher in pregnant women compared to in non‐pregnant women (4.6 vs. 0.6 breaths min^−1^ min^−1^; *β* 0.616, *p* = .015).

**TABLE 3b:** Data are presented as estimated marginal means (*µ*) of slopes with 95% confidence interval and regression coefficients (*β*) after adjustment for age

	Exercise phase before ventilatory threshold						Exercise phase after ventilatory threshold						Recovery phase (1 min)					
	P		NP		*β*	*p*‐value	P		NP		*β*	*p*‐value	P		NP		*β*	*p*‐value
	*µ*	95% CI	*µ*	95% CI			*µ*	95% CI	*µ*	95% CI			*µ*	95% CI	*µ*	95% CI		
V_E_ (L min^−1^ min^−1^)	**6.2**	**5.4; 7.0**	**3.2**	**2.4; 3.9**	**0.861**	**<.001**	10.6	8.0; 13.2	7.4	4.9; 9.8	0.372	.166	−14.2	−20.4; −7.9	−12.3	−17.9; −6.7	−0.098	.729
BF(breaths min^−1^ min^−1^)	−0.9	−2.5; 0.8	0.3	−1.3; 1.8	−0.215	.434	**4.6**	**2.8; 6.4**	**0.6**	**−1.1; 2.3**	**0.616**	**.015**	−5.9	−9.3; −2.5	−1.4	−4.4; 1.6	−0.405	.127
V_t_ (L/min)	**0.4**	**0.3; 0.5**	**0.1**	**0.1; 0.2**	**0.893**	**<.001**	0.2	0.0; 0.3	0.3	0.1; 0.4	−0.230	.400	**−0.2**	**−0.4; 0.0**	**−0.5**	**−0.7; −0.4**	**0.571**	**.036**
VO_2_ (ml min^−1^ min^−1^)	**332**	**288; 375**	**244**	**202; 286**	**0.581**	**.028**	222	151; 293	215	146; 283	0.034	.903	−797	−986; −608	−834	−1002; −666	0.058	.833
VO_2_ (ml kg^−1^ min^−1^ min^−1^)	4.5	3.7; 5.2	4.1	3.4; 4.8	0.149	.587	3.0	1.7; 4.2	3.7	2.5; 4.9	−0.172	.529	−10.7	−13.7; −7.8	−13.6	−16.2; −11.0	0.294	.263
VCO_2_ (ml min^−1^ min^−1^)	**227**	**201; 253**	**147**	**122; 172**	**0.794**	**.001**	345	273; 417	292	223; 361	0.229	.403	−576	−782; −369	−606	−789; −423	0.046	.869
P_ET_O_2_ (mmHg/min)	−4.5	−6.1; −2.9	−6.1	−7.7; −4.6	0.313	.251	4.4	2.8; 6.0	2.8	1.3; 4.4	0.295	.282	10.8	7.4; 14.3	14.8	11.7; 17.8	−0.325	.192
P_ET_CO_2_ (mmHg/min)	1.7	1.1; 2.3	2.6	2.0; 3.2	−0.429	.113	0.3	−0.6; 1.3	0.9	0.0; 1.8	−0.182	.508	−4.3	−6.5; −2.1	−6.7	−8.6; −4.8	0.314	.210
EqO_2_ (min^−1^)	−4.1	−5.7; −2.6	−4.5	−6.1; −3.0	0.081	.763	3.3	2.1; 4.6	2.3	1.1; 3.5	0.230	.365	15.1	10.4; 19.8	16.5	12.3; 20.7	−0.095	.736
EqCO_2_ (min^−1^)	−2.6	−3.4; −1.7	−2.9	−3.7; −2.1	0.124	.651	−0.4	−1.4; 0.6	−0.7	−1.7; 0.2	0.100	.707	4.7	2.9; 6.5	6.2	4.6; 7.9	−0.257	.338

Abbreviations: BF, breathing frequency; EqCO_2_, equivalent of carbon dioxide; EqO_2_, equivalent of oxygen; L, liter; min, minute; mL, milliliter; mmHg, millimeters of mercury; NP, non‐pregnant; P, pregnant; P_ET_CO_2_, end‐tidal pressure of carbon dioxide; P_ET_O_2_, end‐tidal pressure of oxygen; VCO_2_, carbon dioxide production; V_E_, minute ventilation; VO_2_, oxygen consumption; V_t_, tidal volume.

Bold indicates statistical significant values.

Finally, the V_E_/VCO_2_ slope was steeper in pregnant women compared to in non‐pregnant women (29.5 ± 3.6 vs. 25.6 ± 2.2, *p* = .013), while the Oxygen Uptake Efficiency Slope (OUES) did not differ between groups (1899 ± 453 vs. 2,267 ± 390 L/min/log(V_E_), *p* = .428).

#### 3.3.3 Recovery phase

One minute after the exercise phase ended, the respiratory parameters of neither the pregnant nor the non‐pregnant women had returned to their baseline values (Table 3a; Figure 3). At this point, there were no differences in means of V_E_, VO_2_, VCO_2_, P_ET_CO_2_, EqO_2_ or EqCO_2_ between the groups. However, pregnant women had again lower BF values as well as higher V_t_ and P_ET_O_2_ levels compared to non‐pregnant women.

There were no differences in slopes during one minute of recovery between both groups, except for V_t_; pregnant women showed a less steep decrease in V_t_ compared to non‐pregnant women (−0.2 vs. −0.5 L/min; *β* 0.571, *p* = .036; Table 3b).

After 3 min of rest, only P_ET_CO_2_ was lower in the pregnant women, compared to in the non‐pregnant women (26 [95% CI 24; 28] vs. 29 [95% CI 28; 31] mmHg; *p* = .047; Table 3a; Figure 3).

## 4 DISCUSSION

In this pilot study, we examined the effects of early pregnancy on the hemodynamic and respiratory response during CPET in healthy women. In our study population, the hemodynamic response to submaximal exercise testing showed similar slopes of CO, HR, and SV between pregnant and non‐pregnant women. This is in line with the previous work of Ueland et al. and Guzman and Caplan, who both reported the cardiovascular response to exercise to maintain constant from early gestation onward and similar to that encountered in non‐pregnant individuals (Guzman & Caplan, 1970; Ueland et al., 1969). Ueland et al. assessed 11 pregnant women by stationary upright cycle exercise during pregnancy and in the postpartum period. Following mild exercise protocols, the recovery of cardiovascular function to resting values occurred with equal rapidity during pregnancy and post‐partum. Guzman and Caplan studied eight pregnant subjects monthly from the first trimester onward until three months after delivery, with similar findings. We add a greater number of studied women to these findings, assessed by a modern, non‐invasive impedance technique permitting continuous monitoring to assess hemodynamic parameters during incremental exercise to a personalized cycling endpoint (i.e., 70% of the estimated maximum HR). Although not statistically significant, we observed less recovery during one minute of rest following our RAMP protocol in pregnant women compared to non‐pregnant women, indicated by less steep slopes of CO and HR recovery. Hereby we assume the longer time to full recovery of hemodynamic parameters in our pregnant population to be an indicator of less compensatory reserves during early pregnancy due to increased SV and CO in rest (Cornette et al., 2014). Also, HR recovery is a reflection of autonomic function, which is known to be altered during late pregnancy (Steinback et al., 2019).

We observed an increased ventilatory response to exercise in the pregnant group: first, pregnant women showed a much steeper V_E_ slope before the ventilatory threshold compared to non‐pregnant women. Second, the observed higher ventilatory equivalents in pregnant women indicate less ventilatory efficiency in pregnancy, as more ventilation is needed for O_2_ uptake and CO_2_ elimination. Finally, the V_E_/VCO_2_ slope was steeper in pregnant women compared to non‐pregnant women, indicating an excessive rise in V_E_, in relation to CO_2_ production. Especially at 70% of the estimated maximum heart rate, this excessive ventilatory response was evident, with lower P_ET_O_2_ and higher EqCO_2_ in the pregnant women, indicating increased dead space ventilation. Together with similar increases of SV and CO during exercise, an increased ventilation‐perfusion mismatch is observed during exercise in early pregnancy. During exercise, additional oxygen is needed for energy production. With the already increased, resting metabolic demands during early pregnancy (20 kcal/day extra, after adjustment for an increase in body weight), the observed, excessive increase of ventilation is probably necessary (Butte & King, 2005; Most et al., 2019). We did not find evidence for an increased, resting metabolic rate during early pregnancy within our data (i.e., respiratory exchange ratio and VO_2_ levels). It might be that the reference phase of our CPET‐protocol was too short to establish a sufficient steady‐state environment to find such a small increase in resting metabolic rate during the first trimester of pregnancy.

The described, excessive ventilatory response is in accordance with previous studies in early pregnancy (Spatling et al., 1992; Weissgerber et al., 2006) and in late pregnancy (Davenport et al., 2009; Heenan et al., 2001; Jensen et al., 2010; Wolfe et al., 1994). However, we used a modern breath‐by‐breath technique, whereas Spätling et al. measured gas exchange and ventilation parameters by flow‐weighted analysis of mixed, expired gas concentrations, providing 30‐s averages, which therefore is less suitable when rapid changes (e.g., during exercise) are expected. Weissgerber et al. did use a breath‐by‐breath technology—however, in smaller sample size—without information on the ventilatory equivalents or end‐tidal values for O_2_ and CO_2_, and without the assessment of the ventilatory threshold during pregnancy.

Additionally, in our cohort, the pregnant women had lower P_ET_CO_2_ values and higher P_ET_O_2_ values during exercise, with similar rates of change per minute (slopes) for these parameters. This suggests lower arterial blood CO_2_ (P_a_CO_2_) and higher arterial blood O_2_ (P_a_O_2_) levels during rest in early pregnancy, without an effect in response to exercise. Most likely, this is a reflection of the need for a sufficient gradient across the placenta to facilitate efficient gas exchange with the fetus (Wolfe et al., 1998).

The O_2_ uptake in ml/min at the ventilatory threshold was similar in both groups in our study; however, the time to reach this threshold was slightly shorter in pregnant women compared to non‐pregnant women. Since we used a standardized RAMP protocol in both groups, the ventilatory threshold occurred at lower cycle load levels during pregnancy. This is a new finding in human studies, but has been reported earlier in pregnant rats, which also reached their ventilatory threshold at lower exercise intensity levels (Netto et al., 2017). This suggests that during pregnancy, either the aerobic system is used less efficiently or the aerobic system is already used at higher levels during rest, which fits the hypothesis that pregnancy can be considered as a 24‐hr/9‐month lasting mild to moderate exercise. In addition, the combination of the observed steeper slope of BF after the ventilatory threshold with lower P_ET_CO_2_ levels at the ventilatory threshold could be due to a lower ventilatory recruitment threshold for CO_2_, which has been previously described (Jensen et al., 2008).

### 4.1 Strengths and limitations

To our knowledge, only one previous study evaluated a comparable set of hemodynamic and respiratory parameters during exercise in the first trimester of pregnancy (Spatling et al., 1992). With most information on the response to exercise during early pregnancy originating from the previous century, re‐evaluation by modern, non‐invasive techniques with continuous monitoring provides the opportunity to progress in this field of research with possible new physiological insights. Although we are not the first to examine the combined cardiopulmonary response to exercise in early pregnancy, we have included a larger number of women in their first trimester and used modern, non‐invasive techniques to compile a complete set of parameters. By doing so, we were able to confirm and further develop the historical findings on the exercise physiology of pregnancy. We have hereby shown the feasibility of this standardized CPET setup. Reliability and validity tests are now further required before clinical implementation.

However, several limitations should be noted. The main limitation of our study is the observed difference in baseline characteristics between the pregnant and non‐pregnant women, which could have affected CPET outcome (Wasserman et al., 2012). The pregnant women were older compared to the non‐pregnant women, which is why we adjusted all results for age in our statistical analysis. By evaluating the ventilatory threshold as a specific time point and OUES and V_E_/VCO_2_ slope as submaximal parameters, the established differences most likely truly reflect the effect of pregnancy on the response to exercise, regardless of age. Also, although the observed mean BMI in the pregnant group was higher than in the non‐pregnant group and the non‐pregnant subjects reported a higher physical activity level (based on one brief question), we performed two separate regression analyses, which showed that neither the BMI nor physical activity level was a confounder in our dataset (data not shown). When O_2_ uptake was expressed in mL/kg/min, even larger differences between pregnant and non‐pregnant women were observed (Table 3a). This indicates that with a better BMI‐matched control group, similar or even more evident differences are to be expected. Additionally, we analyzed a second, non‐pregnant, age‐ and BMI‐matched control group, collected from BeLife Clinical Human Performance Center (BeLife Health, Rotterdam, the Netherlands), where CPETs are performed for various indications (rehabilitation, improvement of physical condition, upon request of an employer, etc.) using the same equipment. The results of this comparison show similar results as described in this manuscript (data not shown, peer‐reviewed). However, this control group also has some limitations. First, at BeLife, CPETs were performed until exhaustion, so the recovery phase is incomparable. Second, information on V_t_, P_ET_O_2_, and EqO_2_ was lacking, which made it impossible to assess the ventilatory threshold by the ventilatory equivalents method. Third, at baseline, blood pressure was higher in the BeLife group, possibly indicating a less optimal physical condition compared to the pregnant women.

Also, in this pilot study, the number of participants might have been a limitation. However, a post hoc power calculation—based on the response of VO_2_ to exercise and the number of participants—showed that we also had sufficient power (>0.90) to establish differences in our secondary outcomes.

### 4.2 Conclusion and future perspectives

In conclusion, this study confirms that women who are in their first trimester of pregnancy already have an altered response to exercise compared to non‐pregnant women. The observed hemodynamic response to exercise was similar to the response outside pregnancy and a larger increase of ventilation was observed during submaximal CPET. These findings show the need for physiological cardiopulmonary adaptation to pregnancy in women and we hypothesize that any maladaptation to pregnancy could be involved in the pathophysiology of adverse pregnancy outcomes. CPET is a scientifically proven method to assess multiple organ systems during physical stress and can, therefore, be of use to diagnose maladaptation in early pregnancy. We recommend further research using CPET from the preconception period onward, preferably in the same women, to evaluate its use in cardiovascular risk assessment in relation to pregnancy course and outcome (Bijl, Cornette, et al., 2019). Also, resting metabolic requirements in early pregnancy is an interesting topic for future research, although this requires a well‐controlled, steady‐state environment.

## CONFLICT OF INTEREST

None declared.

## AUTHOR CONTRIBUTIONS

The rationale and design of the study were conceived by JMJC, RPMST, and MPHWK. RCB, KvdH, and MLdZ were involved in the acquisition and analysis of the data. Interpretation of the results was performed by RCB, KvdH, MLdZ, and DDRM supervised by JMJC, AF, JM, and MPHWK. RCB prepared the first draft of the manuscript. All authors have critically reviewed and approved the final manuscript and agree to be accountable for all aspects of the work.

## Data Availability

The data that support the findings of this study are available from the corresponding author upon reasonable request.
